# Patients with stage IA ovarian clear cell carcinoma do not require chemotherapy following surgery

**DOI:** 10.1002/cam4.5453

**Published:** 2022-11-23

**Authors:** Li Shuqing, Zhu Zhiling

**Affiliations:** ^1^ Department of Obstetrics and Gynecology Obstetrics and Gynecology Hospital of Fudan University Shanghai China

**Keywords:** chemotherapy, clear cell carcinoma, ovarian cancer, overall survival

## Abstract

**Background:**

Ovarian clear cell carcinoma (OCCC) is an infrequent histological subtype of epithelial ovarian cancer (EOC). The present study aimed to investigate whether chemotherapy is indispensable for patients with stage IA OCCC.

**Methods:**

Data were collected from the Surveillance, Epidemiology and End Results database between 2004 and 2015. All subjects were diagnosed with stage IA OCCC, according to their postoperative pathological reports. In the present study, 1038 patients were retrospectively investigated, among whom 692 patients received chemotherapy. Propensity score matching (PSM) was performed to prevent selection bias. The multivariate Cox proportional hazards model was used to analyze the correlation between variables and 5‐year overall survival.

**Results:**

An equal number of patients (*n* = 346) who did or did not undergo chemotherapy after PSM were further enrolled in the study. The results showed that the mortality of OCCC increased for the patients aged ≥50 years. In addition, older age was associated with lower 5‐year overall survival (*p* < 0.05). However, chemotherapy did not extend the 5‐year overall survival (*p* = 0.524) of patients with stage IA OCCC, according to the multivariate Cox regression analysis.

**Conclusions:**

Chemotherapy did not affect the overall survival of patients with stage IA OCCC following surgery.

## Graphical abstract

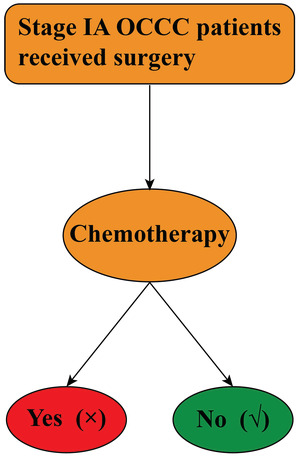

## 1 INTRODUCTION

Epithelial ovarian cancer (EOC) is a highly lethal carcinoma, the global 5‐year overall survival rate of which has stayed at 30%–40% for decades. The standard treatment recommended by the National Comprehensive Cancer Network guidelines is comprehensive surgical staging followed by platinum‐based chemotherapy.^1^, ^2^ However, disease recurrence and chemoresistance pose major challenges to therapeutic efficacy, and contribute to the high mortality rate.^3^, ^4^ Ovarian clear cell carcinoma (OCCC) is an infrequent histological subtype of EOC, which accounts for a variable 5%–25% of all cases of EOC. It has different biologic behavior and clinicopathological characteristics from those of other subtypes of EOC.^5^, ^6^ To be exact, most OCCC are negative with WT1 transcription factor and estrogen receptors. Conversely, high‐grade serous carcinoma is usually positive. OCCC shows frequent phosphatidylinositol‐4,5‐bisphosphate 3‐kinase catalytic subunit alpha mutations and PTEN inactivation, while tumor protein 53 and breast cancer 1/2 mutations are ubiquitous in high‐grade serous carcinoma.^7^ Furthermore, OCCC may arise from ovarian endometriosis and usually presents at a younger age and earlier stage in comparison with high‐grade serous carcinoma. Early OCCC tends to be associated with a more favorable prognosis than late OCCC.^8^, ^9^ However, OCCC is considered to be a high‐grade tumor, and advanced stage OCCC is associated with a poor prognosis due to its intrinsic resistance.^10^, ^11^ Although postoperative treatment is the standard treatment regimen for OCCC,^12^ its overall efficacy for early stage disease remains unclear.^13^ In addition to the insensitivity of OCCC to chemotherapy, one major cause is that OCCC is frequently analyzed concomitantly with other histological subtypes due to its rarity.^14^ For that reason, detailed and integrated information regarding the treatment and prognosis of early OCCC is difficult to acquire and, therefore, scarce.^15^ Subtype‐specific data are thus required if therapeutic strategies for early OCCC are to be optimized in the future.

The present study provided specific data from the US Surveillance, Epidemiology and End Results (SEER) database, which is devoted to improving data quality by performing rigorous quality control studies and various data assessments. Patients with stage IA OCCC were incorporated into our study cohort. All patients received surgery and their follow‐up data were retrospectively analyzed. This study aimed to determine whether adjuvant therapy should be used for the treatment of stage IA OCCC.

## 2 METHODS

### 2.1 Data sourcing

Data were extracted from the SEER database.

### 2.2 Inclusion criteria

**FIGURE 1:**
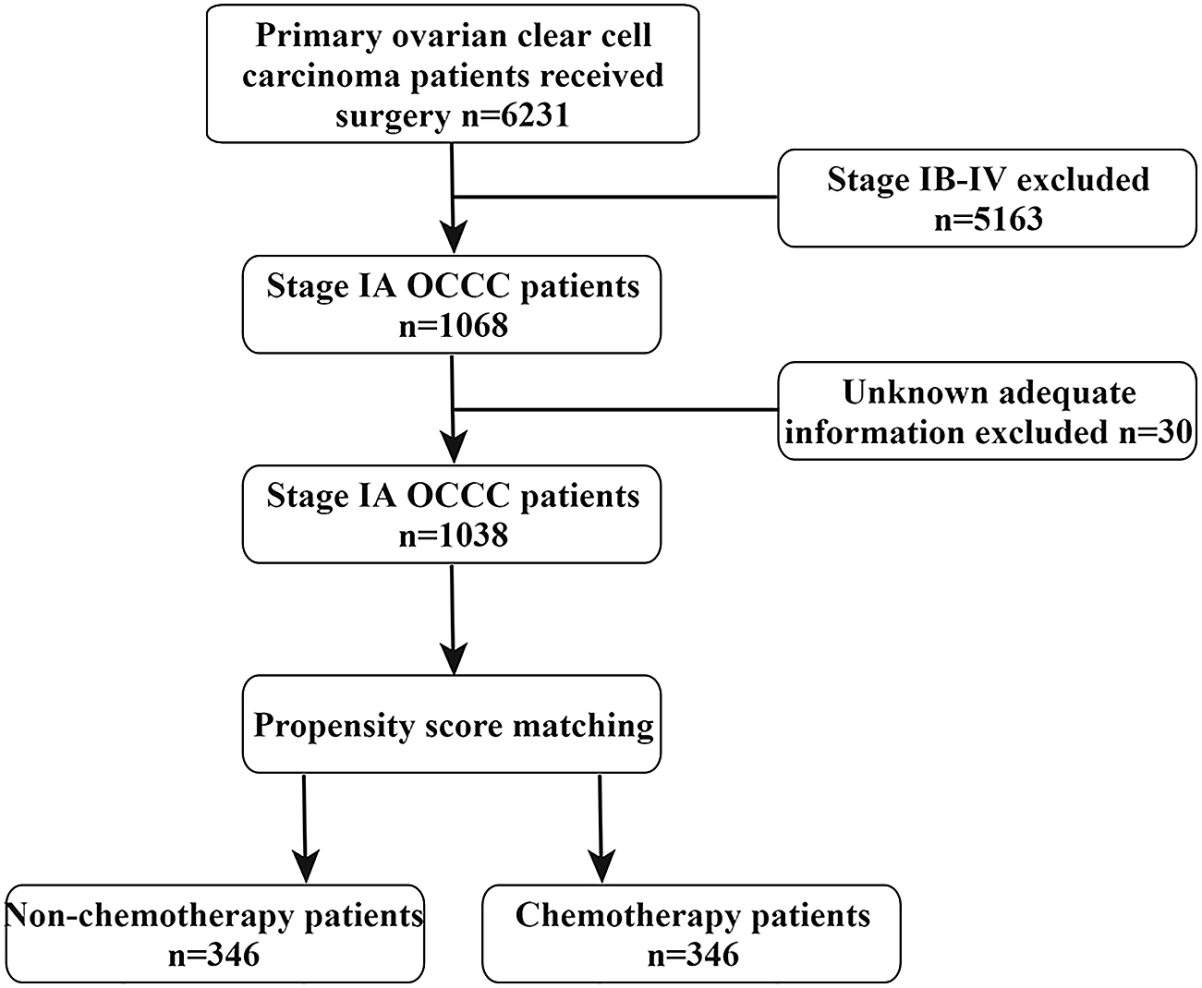
Flow chart.

Patients with primary OCCC (ICD‐O‐3, 8310/3) between 2004 and 2015 were identified and all subjects underwent surgery (*n* = 6231). Data from a total of 1038 patients with stage IA OCCC (Derived AJCC Stage Group, 6th ed) were collected for the present study. The following variables were included and presented as numbers and percentages: Patient identity card, Age at diagnosis, Ethnicity record, Tumor stage (Derived AJCC Stage Group, 6th ed), Laterality (origin of primary), CS (collaborative staging) tumor size, Regional lymph nodes removed, Primary surgical site (ovary), Surgical type, Chemotherapy recode, Survival months, Vital status (dead or alive) and SEER cause‐specific death classification. The subjects were classified into three age groups: <50, 50–60 and > 60 years. Ethnicity was divided into Caucasian and non‐Caucasian. Tumor laterality was grouped into whether the tumor originated from the left or the right ovary. Tumor size was classified into the following three groups: ≤2 cm, >2 cm and unknown. The lymph nodes removed were categorized into ≤3 and ≥4. In the extracted dataset, chemotherapy recode was divided into yes and no/unknown. The effect of chemotherapy on the 5‐year overall survival was assessed for the subjects (Figure 1).

### 2.3 Statistical analysis

#### 2.3.1 Univariate analysis

The follow‐up data of each subject was integrated. Pearson's *χ* ^2^‐tests were used to assess the univariate associations. Two‐sided test was conducted for all statistical tests. *p* < 0.05 was considered statistically significant. Statistical analysis was performed using R and SPSS software.

#### 2.3.2 PSM

Selection bias, which is common in retrospective studies, existed in our research and might affect the results of the present study. To minimize the influence of selection bias, PSM and logistic regression models were used to match age, ethnicity, tumor laterality, tumor size and number of regional lymph nodes removed between the chemotherapy and non‐chemotherapy groups.

### 2.4 Survival analysis

The Kaplan–Meier method was applied to perform survival analysis. The hazard ratios (HRs) and 95% confidence intervals (CIs) were evaluated using the Cox proportional hazards model.

## 3 RESULTS

### 3.1 Demographic features

A total of 1038 patients (346 who had not received chemotherapy and 692 who had) met the study inclusion criteria. Among these patients, 26.2% were aged <50 years, 43.0% were aged 50–60 years and 30.8% were aged ≥60 years. The majority of patients were Caucasian (*n* = 823; 79.3%). An equal number of left‐ (*n* = 519; 50.0%) and right‐sided (*n* = 519; 50.0%) cases were observed in terms of tumor laterality. Only 8.0% of the patients had tumors measuring ≤2 cm in size, whereas 79.5% of the patients had tumors measuring >2 cm. In total, ≤3 lymph nodes were removed from 28.4% of the patients, whilst ≥4 lymph nodes were removed from 71.6% of the patients (Table 1).

**TABLE 1:** Demographics

Characteristics	No^a^	%
Chemotherapy		
No	346	33.3
Yes	692	66.7
Age		
<50y	272	26.2
50–60y	446	43.0
>60y	320	30.8
Ethnicity		
Caucasian	823	79.3
Non‐Caucasian	215	20.7
Laterality^b^		
Left	519	50.0
Right	519	50.0
Tumor size		
≤2 cm	83	8.0
>2 cm	825	79.5
Unknown	130	12.5
Lymph node		
≤3	295	28.4
≥4	743	71.6

^a^ Data are expressed as *n* (%).

^b^ Laterality: the side of ovaries on which the primary tumor originated.

### 3.2 Comparison of univariate covariates

Compared with patients in the non‐chemotherapy groups, patients in the chemotherapy groups were younger prior to PSM (<50 years, 24.4 vs. 29.8%, respectively; 50–60 years, 46.8 vs. 35.3%, respectively; and > 60 years, 28.8 vs. 35.0%, respectively; *p* < 0.05). The patients were more likely to be Caucasian (79.6 vs. 78.6%, respectively; *p* = 0.705). There was also a higher number of patients in whom the left ovary was the primary origin in the chemotherapy groups (50.3 vs. 49.4%, respectively; *p* = 0.792). Fewer patients had tumors measuring ≤2 cm in the chemotherapy groups (7.8 vs. 8.4%, respectively; *p* = 0.090). Finally, there were fewer patients with ≤3 lymph nodes removed in the chemotherapy groups compared with the non‐chemotherapy groups (24.3 vs. 36.7%, respectively; *p* < 0.05). PSM was performed to eliminate the non‐uniformity between the two groups and equalized the number of patients (*n* = 346). The logistic regression analysis demonstrated that the covariates were well balanced and markedly decreased (Table 2).

**TABLE 2:** Comparison of univariate covariates

Characteristics		Before PSM	*p*		After PSM	*p*
	Chemotherapy − (*n* = 346)	Chemotherapy + (*n* = 692)		Chemotherapy − (*n* = 346)	Chemotherapy + (*n* = 346)	
Age						
<50y	103(29.8)	169(24.4)	<0.01	103(29.8)	104(30.1)	0.996
50–60y	122(35.3)	324(46.8)		122(35.3)	122(35.3)	
>60y	121(35.0)	199(28.8)		121(35.0)	120(34.7)	
Ethnicity						
Caucasian	272(78.6)	551(79.6)	0.705	272(78.6)	267(77.2)	0.647
Non‐Caucasian	74(21.4)	141(20.4)		74(21.4)	79(22.8)	
Laterality						
Left	171(49.4)	348(50.3)	0.792	171(49.4)	163(47.1)	0.543
Right	175(50.6)	344(49.7)		175(50.6)	183(52.9)	
Tumor size						
≤2 cm	29(8.4)	54(7.8)	0.090	29(8.4)	24(6.9)	0.760
>2 cm	263(76.0)	562(81.2)		263(76.0)	269(77.7)	
Unknown	54(15.6)	76(11.0)		54(15.6)	53(15.3)	
Lymph node						
≤3	127(36.7)	168(24.3)	<0.001	127(36.7)	128(37.0)	0.937
≥4	219(63.3)	524(75.7)		219(63.3)	218(63.0)	

Abbreviation: PSM, propensity score matching.

### 3.3 Relevance between chemotherapy and overall survival

The potential relevance between chemotherapy and 5‐year overall survival was analyzed. No statistically significances were identified between the chemotherapy and non‐chemotherapy groups (54.6 vs. 55.8%, respectively; *p* = 0.760). Therefore, patients with stage IA OCCC did not benefit from chemotherapy in the field of 5‐year overall survival (Table 3).

**TABLE 3:** Relevance of chemotherapy with 5‐year overall survival

Chemotherapy	Life	Death	%	*p*
No	193	153	55.8	0.760
Yes	189	157	54.6	

### 3.4 Univariate analysis of clinical factors with overall survival

**FIGURE 2:**
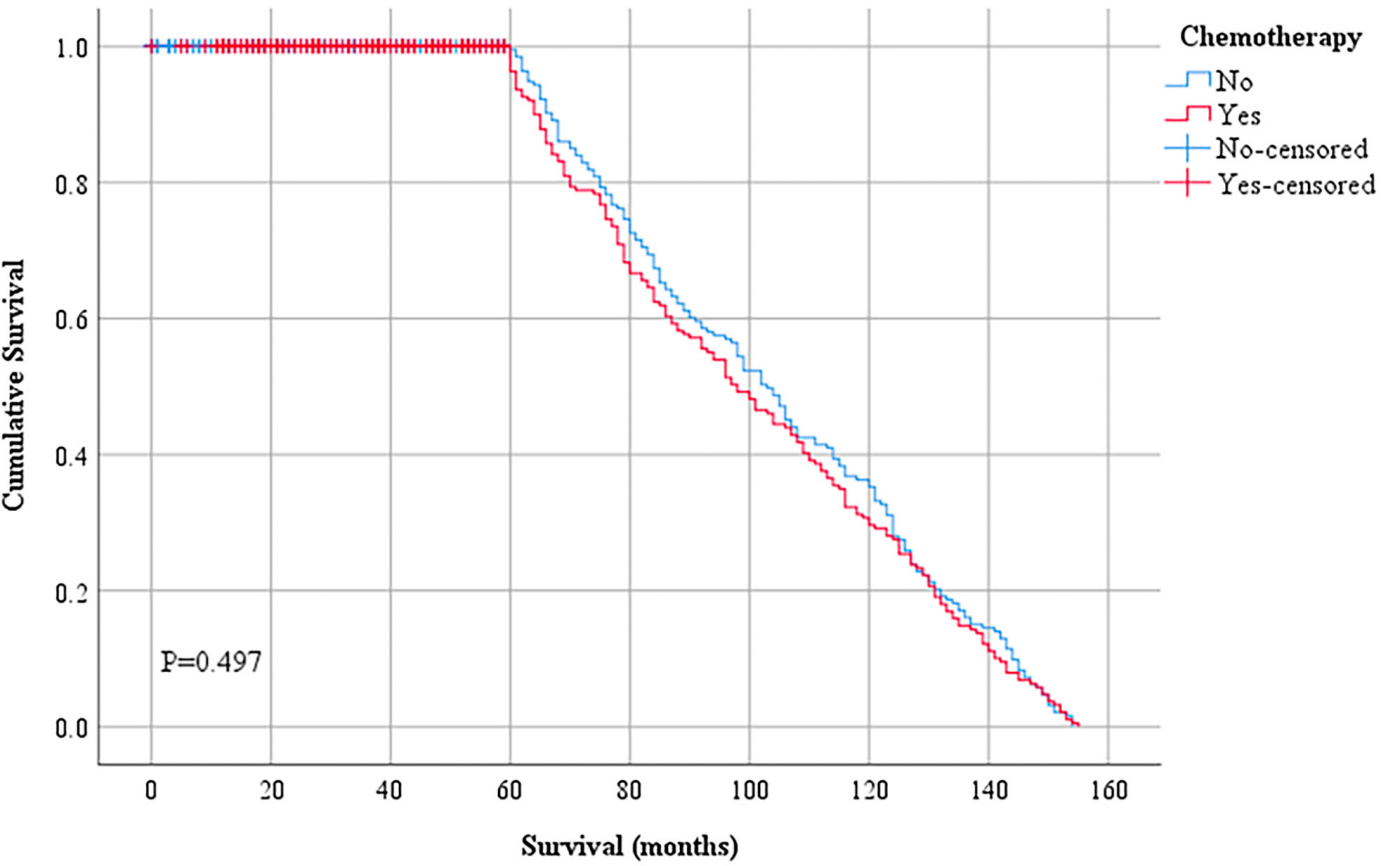
Kaplan–Meier survival curves for 5‐year overall survival in patients with stage IA OCCC after propensity score matching. *p* = 0.497.

A univariate analysis was conducted in the matched patients to study the prognostic effects of the clinical factors (Table 4 and Figure 2). No significant differences in the 5‐year overall survival rates were identified between the chemotherapy and non‐chemotherapy groups (HR, 0.934; 95% CI, 0.764–1.142; *p* = 0.506). Older age was a risk factor for 5‐year overall survival (50–60 years, HR, 0.713; 95% CI, 0.555–0.915; >60 years, HR, 0.750; 95% CI, 0.584–0.963; *p* < 0.05) rates. Tumors >2 cm in size were associated with a lower 5‐year overall survival (HR, 1.545; 95% CI, 0.976–2.446; *p* < 0.05) rates.

**TABLE 4:** Univariate analysis and multivariate cox regression analysis for 5‐year overall survival

Characteristics	Univariate analysis		Multivariate analysis	
	HR (95% CI)	*p*	HR (95% CI)	*p*
Chemotherapy				
No	Ref	0.506	Ref	0.524
Yes	0.934(0.764–1.142)		/	
Age				
<50y	Ref	0.018	Ref	0.021
50–60y	0.713(0.555–0.915)		1.052(0.826–1.339)	
>60y	0.750(0.584–0.963)		1.403(1.093–1.801)	
Ethnicity				
Caucasian	Ref	0.682	Ref	0.326
Non‐Caucasian	0.950(0.746–1.212)		/	
Laterality				
Left	Ref	0.062	Ref	0.084
Right	0.824(0.673–1.010)		/	
Tumor size				
≤2 cm	Ref	0.040	Ref	0.093
>2 cm	1.545(0.976–2.446)		/	
Unknown	1.378(1.060–1.792)		/	
Lymph node				
≤3	Ref	0.460	Ref	0.526
≥4	0.924(0.750–1.139)		/	

Abbreviations: CI, confidence intervals; HR, hazard ratios; Ref: reference.

### 3.5 Cox proportional hazards model

The correlation between variables and survival is extensively investigated using the Cox proportional hazards model. The Kaplan–Meier method and log‐rank tests are used to analyze patient survival according to one factor in isolation, without including the effect of other factors. They are only applicable for categorical variables, not for quantitative or parametric variables. The Cox proportional hazards model is suitable for both categorical and quantitative variables. In addition, the Cox proportional hazards model allows for the simultaneous assessment of the effects of several variables on survival.

To investigate how the variables collectively affect survival, the present study incorporated these variables into the multivariate Cox regression analysis (Table 4). The results suggested that elderly patients (age, ≥50 years) had a higher mortality and a lower 5‐year overall survival (50–60 years, HR, 1.052; 95% CI, 0.826–1.339; >60 years, HR, 1.403; 95% CI, 1.093–1.801; *p* < 0.05) rates. After eliminating the effect of miscellaneous factors, however, chemotherapy remained statistically insignificant in the field of the 5‐year overall survival (*p* = 0.524).

## 4 DISCUSSION

OCCC accounts for 3%–11% of all EOC cases in the United States.^16^, ^17^ Staging surgery or cytoreductive surgery combined with chemotherapy is the current standardized treatment for OCCC, and clinical guidelines recommend standard intravenous platinum‐based chemotherapy for patients with stage I‐IV OCCC,^18^ though it is characterized as inherent chemoresistance and poor prognosis.^19^, ^20^ However, the effect of adjuvant chemotherapy in early OCCC remains the subject of intense debate on account of the rarity of the disease and the lack of large sample clinical trials.^21^

The strength of the present study was that it provided specific and powerful evidence on the efficacy of adjuvant chemotherapy for treating stage IA OCCC because the cohort was based on a great and distinct population. However, all SEER database analyses have inherent limitations, which may have resulted in important biases in the present study.^22^ Precise surgical data were arduous to acquire for the subjects. In addition, this dataset lacked information regarding the details of adjuvant chemotherapy, including the number of cycles, agents and dosage.^23^ To weaken selection bias, the dataset was randomized and causal arguments were strengthened using PSM. The Cox proportional‐hazards models were performed to analyze the correlation between variables and survival.

No significant differences in the 5‐year overall survival were discovered between the chemotherapy and non‐chemotherapy groups (*p* = 0.497). The multivariate Cox regression analysis showed that elderly patients (age, ≥50 years) had a higher mortality and a lower 5‐year overall survival (*p* < 0.05). After eliminating the effect of miscellaneous factors, it was observed that the difference between chemotherapy and the 5‐year overall survival was not statistically significant (*p* = 0.524).

The present findings were consistent with those of previous studies. A retrospective investigation suggested that adjuvant chemotherapy had little effect on the survival of stage I OCCC patients (*n* = 219), and no statistical difference between progression‐free and overall survival was identified.^24^ Similarly, Mizuno et al found that postoperative adjuvant chemotherapy was not helpful in improving the prognosis of patients with stage IA OCCC (*p* = 134). There was no significant prognostic factor for either recurrence or survival.^25^ Equally, Takada et al revealed that postoperative adjuvant chemotherapy did not influence progression‐free and overall survival in patients with stage IA CCC (*n* = 20).^26^ By the same token, Oseledchyk et al evaluated the impact of adjuvant chemotherapy on overall survival in patients with stage I OCCC using the SEER database. Their analysis indicated that chemotherapy was not associated with improved overall survival (*n* = 1995).^27^ Another retrospective study demonstrated that postoperative adjuvant chemotherapy was not associated with overall survival, and could be safely omitted in patients with stage IA and IB OCCC (*n* = 45).^28^ In addition, a meta‐analysis revealed that adjuvant chemotherapy did not affect disease‐free survival and overall survival in patients with stage IA and IB OCCC (*n* = 2809).^29^ However, the present findings were inconsistent with those of a study by Hogen et al, which supported the use of adjuvant chemotherapy for surgical stage I OCCC (*n* = 60).^30^ Furthermore, a cohort of postoperative patients with stage I OCCC (1298 patients with stage IA, 35 patients with stage IB, and 1007 patients with stage IC) was drawn from the National Cancer Database. That study indicated that adjuvant chemotherapy was associated with a better overall survival.^31^ The discordance in findings could be ascribed to several factors, including differences in patient population, surgical protocols, chemotherapy paradigm, tumor misclassifications, or tumor heterogeneity.

In conclusion, the present investigation suggested that the adoption of adjuvant chemotherapy does not improve the outcomes of patients with stage IA OCCC after surgery has been performed. However, convincing evidences from large‐scale clinical trials are needed to confirm this encouraging outcome. Further study is required to provide guidance on the optimal management of patients with early OCCC.

## AUTHOR CONTRIBUTIONS

**Shuqing Li:** Conceptualization (lead); data curation (lead); formal analysis (lead); investigation (lead); methodology (lead); project administration (lead); resources (lead); software (lead); writing – original draft (lead); writing – review and editing (lead).

## CONFLICTS OF INTEREST

The authors declare that they have no conflicts of interest.

## Data Availability

The datasets analyzed during the present study are available from the corresponding author on reasonable request.

## References

[1] Zhang J , Guan W , Xu X , Wang F , Li X , Xu G . A novel homeostatic loop of sorcin drives paclitaxel‐resistance and malignant progression via Smad4/ZEB1/miR‐142‐5p in human ovarian cancer. Oncogene. 2021;40(30):4906‐4918. 34163033 10.1038/s41388-021-01891-6 PMC8321900

[2] Wu PY , Cheng YM , Shen MR , Chen YC , Huang YF , Chou CY . Real‐world study of adding bevacizumab to chemotherapy for ovarian, tubal, and peritoneal cancer as front‐line or relapse therapy (ROBOT): 8‐year experience. Front Oncol. 2020;10:1095. 32760668 10.3389/fonc.2020.01095 PMC7372289

[3] Karimnia N , Wilson AL , Green E , et al. Chemoresistance is mediated by ovarian cancer leader cells in vitro. J Exp Clin Cancer Res. 2021;40(1):276. 34470672 10.1186/s13046-021-02086-3 PMC8408956

[4] Alharbi M , Lai A , Sharma S , et al. Extracellular vesicle transmission of chemoresistance to ovarian cancer cells is associated with hypoxia‐induced expression of glycolytic pathway proteins, and prediction of epithelial ovarian cancer disease recurrence. Cancers (Basel). 2021;13(14):3388. 34298602 10.3390/cancers13143388 PMC8305505

[5] Prodromidou A , Theofanakis C , Thomakos N , Haidopoulos D , Rodolakis A . Fertility sparing surgery for early‐stage clear cell carcinoma of the ovary; a systematic review and analysis of obstetric outcomes. Eur J Surg Oncol. 2021;47(6):1286‐1291. 33509613 10.1016/j.ejso.2021.01.016

[6] Su KM , Lin TW , Liu LC , et al. The potential role of complement system in the progression of ovarian clear cell carcinoma inferred from the gene ontology‐based Immunofunctionome analysis. Int J Mol Sci. 2020;21(8):2824. 32316695 10.3390/ijms21082824 PMC7216156

[7] McCluggage WG , Judge MJ , Clarke BA , et al. Data set for reporting of ovary, fallopian tube and primary peritoneal carcinoma: recommendations from the international collaboration on cancer reporting (ICCR). Mod Pathol. 2015;28(8):1101‐1122. 26089092 10.1038/modpathol.2015.77

[8] Yin S , Gao W , Shi P , Xi M , Tang W , Zhang J . Primary laparoscopic surgery does not affect the prognosis of early‐stage ovarian clear cell cancer. Cancer Manag Res. 2021;13:6403‐6409. 34421313 10.2147/CMAR.S321173 PMC8372305

[9] Lee HY , Hong JH , Byun JH , et al. Clinical characteristics of clear cell ovarian cancer: a retrospective multicenter experience of 308 patients in South Korea. Cancer Res Treat. 2020;52(1):277‐283. 31319640 10.4143/crt.2019.292 PMC6962489

[10] Kondo E , Tabata T , Suzuki N , et al. The post‐progression survival of patients with recurrent or persistent ovarian clear cell carcinoma: results from a randomized phase III study in JGOG3017/GCIG. J Gynecol Oncol. 2020;31(6):e94. 33078599 10.3802/jgo.2020.31.e94 PMC7593225

[11] Zhao T , Shao Y , Liu Y , Wang X , Guan L , Lu Y . Endometriosis does not confer improved prognosis in ovarian clear cell carcinoma: a retrospective study at a single institute. J Ovarian Res. 2018;11(1):53. 29941051 10.1186/s13048-018-0425-9 PMC6019519

[12] Ceppi L , Grassi T , Galli F , et al. Early‐stage clear cell ovarian cancer compared to high‐grade histological subtypes: An outcome exploratory analysis in two oncology centers. Gynecol Oncol. 2021;160(1):64‐70. 33077259 10.1016/j.ygyno.2020.10.014

[13] Roy S , Hoskins P , Tinker A , Brar H , Bowering G , Bahl G . Adjuvant treatment of early ovarian clear cell carcinoma: a population‐based study of whole abdominal versus pelvic nodal radiotherapy. J Natl Compr Canc Netw. 2020;19(2):172‐180. 32971514 10.6004/jnccn.2020.7609

[14] Kucukgoz Gulec U , Paydas S , Guzel AB , Vardar MA , Khatib G , Gumurdulu D . The clinical characteristics and outcomes of cases with pure ovarian clear cell, mixed type and high‐grade serous adenocarcinoma. Arch Gynecol Obstet. 2015;292(4):923‐929. 25855053 10.1007/s00404-015-3699-9

[15] Wang T , Zeng J , Li N , Zhang R , Song Y , Wu L . An exploratory analysis about cycles of adjuvant chemotherapy and outcomes by substage for stage I ovarian clear cell carcinoma: a single institution retrospective study. Am J Cancer Res. 2020;10(12):4561‐4567. 33415019 PMC7783737

[16] Ukai M , Suzuki S , Yoshihara M , et al. Adjuvant taxane plus platinum chemotherapy for stage I ovarian clear cell carcinoma with complete surgical staging: are more than three cycles necessary? Int J Clin Oncol. 2022;27(3):609‐618. 34779959 10.1007/s10147-021-02075-8

[17] Zhu C , Zhu J , Qian L , et al. Clinical characteristics and prognosis of ovarian clear cell carcinoma: a 10‐year retrospective study. BMC Cancer. 2021;21(1):322. 33766002 10.1186/s12885-021-08061-7 PMC7993454

[18] Armstrong DK , Alvarez RD , Bakkum‐Gamez JN , et al. Ovarian cancer, version 2.2020, NCCN clinical practice guidelines in oncology. J Natl Compr Canc Netw. 2021;19(2):191‐226. 33545690 10.6004/jnccn.2021.0007

[19] Tate S , Nishikimi K , Matsuoka A , et al. Bevacizumab in first‐line chemotherapy improves progression‐free survival for advanced ovarian clear cell carcinoma. Cancers (Basel). 2021;13(13):3177. 34202220 10.3390/cancers13133177 PMC8268306

[20] Sun Y , Liu G . Endometriosis‐associated ovarian clear cell carcinoma: a special entity? J Cancer. 2021;12(22):6773‐6786. 34659566 10.7150/jca.61107 PMC8518018

[21] Chen Q , Wang S , Lang JH . Clinical characteristics and prognostic factors of stage IC ovarian clear cell carcinoma: a surveillance, epidemiology, and end results (SEER) analysis. Arch Gynecol Obstet. 2021;304(2):521‐529. 33543331 10.1007/s00404-020-05952-1

[22] Matsuo K , Machida H , Matsuzaki S , et al. Evolving population‐based statistics for rare epithelial ovarian cancers. Gynecol Oncol. 2020;157(1):3‐11. 31954534 10.1016/j.ygyno.2019.11.122 PMC7526050

[23] Zhu Y , Meng F , Fang H , Zhang Z , Wang L , Zheng W . Clinicopathologic characteristics and survival outcomes in neuroendocrine carcinoma of the ovary. Int J Gynecol Cancer. 2020;30(2):207‐212. 31796530 10.1136/ijgc-2019-000746

[24] Takano M , Sugiyama T , Yaegashi N , et al. Less impact of adjuvant chemotherapy for stage I clear cell carcinoma of the ovary: a retrospective Japan clear cell carcinoma study. Int J Gynecol Cancer. 2010;20(9):1506‐1510. 21119366 10.1111/IGC.0b013e3181fcd089

[25] Mizuno M , Kajiyama H , Shibata K , et al. Adjuvant chemotherapy for stage i ovarian clear cell carcinoma: is it necessary for stage IA? Int J Gynecol Cancer. 2012;22(7):1143‐1149. 22801028 10.1097/IGC.0b013e31825c7cbe

[26] Takada T , Iwase H , Iitsuka C , et al. Adjuvant chemotherapy for stage I clear cell carcinoma of the ovary: an analysis of fully staged patients. Int J Gynecol Cancer. 2012;22(4):573‐578. 22398705 10.1097/IGC.0b013e31823fd413

[27] Oseledchyk A , Leitao MM Jr , Konner J , et al. Adjuvant chemotherapy in patients with stage I endometrioid or clear cell ovarian cancer in the platinum era: a surveillance, epidemiology, and end results cohort study, 2000‐2013. Ann Oncol. 2017;28(12):2985‐2993. 28950307 10.1093/annonc/mdx525 PMC5834056

[28] Liu SL , Tinker AV . Omission of adjuvant therapy in stage I clear cell ovarian cancer: review of the BC cancer experience. Gynecol Oncol Rep. 2020;31:100533. 31970284 10.1016/j.gore.2019.100533 PMC6965725

[29] Bogani G , Ditto A , Lopez S , et al. Adjuvant chemotherapy vs. observation in stage I clear cell ovarian carcinoma: a systematic review and meta‐analysis. Gynecol Oncol. 2020;157(1):293‐298. 31980220 10.1016/j.ygyno.2019.12.045

[30] Hogen L , Brar H , Covens A , et al. Is adjuvant chemotherapy beneficial for surgical stage I ovarian clear cell carcinoma? Gynecol Oncol. 2017;147(1):54‐60. 28760368 10.1016/j.ygyno.2017.07.128

[31] Nasioudis D , Mastroyannis SA , Albright BB , Haggerty AF , Ko EM , Latif NA . Adjuvant chemotherapy for stage I ovarian clear cell carcinoma: patterns of use and outcomes. Gynecol Oncol. 2018;150(1):14‐18. 29751993 10.1016/j.ygyno.2018.04.567

